# A Central Fund for Provincial Hospitals

**Published:** 1922-08

**Authors:** Gilbert D. Shepherd

**Affiliations:** Secretary. The Prince of Wales' Hospital, Cardiff


*To the Editor of* The Hospital and Health Review.

Yours faithfully,

A. Saxon Snell.

9 Bentinck Street,
Manchester Square, W.1.

### A Central Fund for Provincial Hospitals.

*To the Editor of* The Hospital and Health Review.

Sir,—I have pleasure in stating that at a meeting of the council of this hospital held at No. 10, Downing Street, London, on July 14, under the chairmanship of Dame Margaret Lloyd George, the following resolution was unanimously passed on the proposition of Mr. James Miles, J.P., seconded by Sir John Lynn-Thomas, K.B.E., C.B., C.M.G., viz. :—

"That the Council of the Prince of Wales' Hospital, Cardiff, welcomes the proposal for the establishment of a Central Fund for Provincial Hospitals on the lines of King Edward's Fund for London Hospitals, and begs to express the hope that the said fund may be started at an early date.

"That copies of the foregoing resolution be sent to the British Hospitals Association and to the Editor of The Hospital and Health Review."

Yours faithfully,
Gilbert D. Shepherd,
Secretary.
The Prince of Wales' Hospital, Cardiff.

